# On Diseases Peculiar to Women, Including Displacements of the Uterus

**Published:** 1861-03

**Authors:** 


ART. II.—*On Diseases Peculiar to Women, including Displacements of the Uterus.* By HUGH L. HODGE, M.D., Professor of Obstetrics and Diseases of Women and Children in the University of Pennsylvania. "*Nullius addictus jurare in Verba Magistri.*" With Original Illustrations. 8vo., pp. 442. Philadelphia: Blanchard & Lea, 1860.

UNDER the above rather indefinite title, Dr. Hodge presents us with an exposition of his particular views and practice in respect to a class of diseases, or, perhaps, more strictly speaking, of morbid phenomena incident to the female sex, which have their rise in a state of abnormal irritation—a morbidly excitable condition—of the proper tissue of the nerves of animal life, while those of organic life are so slightly or indirectly involved as to demand but little or no attention whether in a pathological or therapeutical point of view. The most frequent origin of the strictly neurotic ailments alluded to being, according to the experience of the author, a state of morbid irritability of the uterus and its appendages, occurring more especially during the menstrual life of the female.

The pathological views and practical directions embraced in the present treatise are presented by Dr. Hodge as the result of clinical observations collected by him during a long and extensive practice, thirty years of which have been spent in the public teaching of obstetrics and the diseases of women and children.

The morbid phenomena described by Dr. Hodge are unquestionably of very frequent occurrence in the female sex, especially in those individuals who possess naturally an eminently nervous temperament, or in whom that temperament has been developed by a life of luxurious or vicious indulgence. The phenomena referred to form a class of diseases that have attracted much of the attention of the medical profession, in consequence of their obscure and often mimetic character, the strange complication of their symptoms, the peculiar sense of suffering, if not of actual pain, by which they are attended, their resistance to all the ordinary plans of treatment, and their liability speedily to recur after they have been apparently subdued. From this circumstance, as well as from the long experience of its author, his known habits of careful observation and deliberate judgment, and his entire reliability as an accurate and truthful recorder of the facts that have fallen under his notice, the work of Dr. Hodge comes to us clothed with no trifling degree of interest and authority. It is strictly an exposition of the author's own views and practice, and not a summary of the contributions of others upon the subjects discussed, or a critical review of their opinions and plans of treatment.

While, however, we yield all the deference which is justly due to the attainments, character, experience and skill of Dr. Hodge, in the particular department of medicine to which he has devoted chiefly his attention, we are unable to indorse all that he advances in respect at least to the etiology of the particular morbid conditions of which he treats, and to their prophylactic and remedial management. We admit every fact he has adduced, respect his experience, and feel no disposition to doubt the accuracy of his observations. Still, as all are perfectly aware, facts are susceptible of different interpretations; experience the most ample is often fallacious, and the apparently well matured judgment liable to be diverted from just deductions by a habit of regarding things in a single point of view, or through the disturbing influence of a preconceived theory. The work before us is nevertheless, as a whole, one of great ability, and cannot fail to secure for itself a favorable reception from the profession. It is replete with original views, and worthy of a close and candid examination on the part of every practitioner.

After a careful examination of the subject, it will be scarcely possible to doubt the occurrence of a class of diseases dependent solely upon nervous irritation, actual physical maladies resulting from an irritation of the medullary matter of the brain, spinal cord, and their radiations—the nerves; the disturbance of functions thence resulting being confined in many cases to those strictly of animal life. Of these maladies Dr. Hodge is almost the only faithful delineator and historian, while many of the leading practical directions for their prevention and treatment set forth by him will be found, we feel persuaded, when strictly carried into practice, well adapted to the relief of the sufferings by which they are almost invariably attended, and which, in most cases, will prove rebellious to the ordinary plans of practice based upon erroneous views of their nature and their causes.

The treatise is divided into three parts: the *first* treats of diseases of irritation or of exalted and deranged irritability or susceptibility; the *second*, of displacements of the uterus, which Dr. Hodge sets down as the most frequent cause of the diseases treated of in the first part; while the *third* part treats of diseases of sedation, organic and nervous, but more especially of sedation of the uterus, and of its result, amenorrhœa. The fact is, the leading subject of the work throughout is the pathology and therapeutics of the neurotic conditions of the womb and its appendages.

The initial chapter of the first part of the treatise is devoted to a general consideration of the subject of nervous irritation and its consequences; subjects upon which the author entertains views somewhat different from those taught in the books.

The terms *irritability* and *irritation* Dr. Hodge would, if possible, restrict to morbid conditions of the tissues and organs, while the terms *excitability* and *excitement* he would have applied to them only when in their healthy state, as indicative of their susceptibility to the normal excitants and their reaction under their influence.

After pointing out the difference in the amount of excitability—the susceptibility to impressions—possessed by the different tissues and organs of the human frame, at the different periods of life, by the two sexes, and by different individuals of either sex, Dr. Hodge lays down the distinction between *organic* and *nervous irritation*, or in other words, between irritation of the organic and animal life of the organism, between inflammatory and strictly neurotic diseases. He next considers the true character of the pathological condition of the blood-vessels, denominated *congestion*, with the view more especially of drawing the proper distinction between simple nervous congestion, which is often a physiological, healthy occurrence, and inflammatory congestion, which is always a pathological condition.

An increase of the normal secretions of the congested organs is noticed as one of the more constant results of simple, temporary congestion. When, however, the congestion is long continued, or transcends the strictly physiological grade, not only may excessive effusion occur in the closed cavities, producing mischief that is often irremediable, and from, also, the free secretory surfaces, causing great inconvenience and debility, but even effusions of blood within the substance of the organs, giving rise thus to apoplexy of the brain, of the lungs, etc., or from the mucous surfaces, producing hemorrhagic discharges from the lungs, stomach, bowels, uterus, etc.

Congestion sometimes, according to Dr. Hodge, becomes a stimulus to the organic actions of the part in which it is seated, so that the nutritive functions are more actively developed, causing the organ to increase in size, to become *hypertrophied*, but without any change in its tissues, save that of augmented bulk. Inflammation, he believes, never to be the result of simple nervous congestion.

*Neuralgia*, according to our author, is simply the result of an irritation of the proper tissue of a nerve of sensation, while *spasm*, *cramps*, and *tonic* and *clonic convulsions* are the phenomena caused by an irritation of the proper tissue of a motor nerve. But it is not merely by decided pain, spasms, and convulsions that simple nervous irritation is manifested, it may give rise, instead of these, to an innumerable and indescribable variety of morbid sensations and states of feeling, that may be, perhaps, generally speaking, of comparatively trifling import, but which are often of a character the most distressing and terrible, ranging in fact in different cases from a simple *malaise* to a condition of distress and agony that renders life itself scarcely endurable.

Dr. Hodge lays down the general proposition that in the human organism the excitability of any organ is always inversely as its strength. The stronger, more robust, more healthful the individual, the less is his nervous excitability, the less nervous his temperament, the more powerful are the impressions it requires to disturb his mental and physical susceptibilities. Let, however, the strong man be weakened in any way, whether by deprivation of food or sleep, over-exertion, or want of exercise, disease, etc., he will become nervous, his mind will become excitable, and his body keenly susceptible to every impression. As he again regains his strength his irritability diminishes, and he becomes less sensitive, less impressible.

Dr. Hodge defines the hysterical, or neurotic diseases of women, as states of irritation of the cerebro-spinal nervous system in whole or in part. Very evanescent when the cause which produces them is transitory, as in nervous affections from moral impressions, but very persistent in all cases where the cause remains operative; hence the indomitable character of certain nervous or neuralgic affections, from the persistence of their cause, either because it has been entirely overlooked, or, if discovered, found not to be removable.

The task undertaken by Dr. Hodge in the treatise under review is to illustrate the general views, of which we have endeavored, thus, to give a brief outline, by a very full account of the nervous disturbances excited or kept up by the uterus and its appendages when in a state of morbid irritation, and hence to deduce the proper pathology and therapeutics of irritable diseases in women.

The subject thus chosen as the exponent of the neurotic diseases of the female sex, namely, irritable uterus, its symptoms, reflex influences, progress, results, and treatment, with a consideration of the pathology of irritable diseases generally, occupies the eleven remaining chapters of the first part of the treatise.

The history given of the local and general phenomena indicative of irritable uterus is particularly full. Whatever opinion may be entertained as to the true cause of these phenomena, the faithfulness of the author's description of them cannot be called in question.

The local symptoms of irritable uterus, as described in the first part of chapter three, are pain and various distressing sensations, aggravated by motion and by every cause, physical, moral, or mental, which imparts a shock to the womb, or directly or indirectly stimulates it or its appendages. The local morbid phenomena in cases of irritable uterus are usually aggravated by menstruation, sometimes to an intense degree. This constitutes "the *simple dysmenorrhœa*" of Dr. Hodge. Which he defines to be, essentially, a neuralgic and spasmodic affection of the womb, dependent upon the morbidly sensitive—simply neurotic—condition of the tissues involved.

When very intense or long continued, the irritable condition of the uterus may give rise to nervous congestion, as a consecutive or secondary affection. When this happens, besides a sense of fullness, swelling, weight, and bearing down, there will be detected, by a digital examination, a swollen condition of the cervix uteri, as at the beginning of pregnancy, with a divergent state of the lips of the os, showing a tumefaction of the reflected membrane of the vagina, as it becomes uterine, the os uteri being actually or apparently more patulous than is normally the case. This swelling is always greatest during the catamenia, after a long walk, after coition, or, indeed, after any physical or moral excitement. A similar swollen condition may often be perceived at the orifice of the urethra, in its caruncle, in the arborescent and vaginal surface under the urethra, and throughout the vulvo-uterine canal. By the speculum, the mucous membrane of the vulva, vagina, cervix, and os uteri, especially the latter, will be found of a red or scarlet, sometimes of a purplish color. The fluctuating character of this congestion is shown by the varying condition of the parts as to intumescence and coloration, at different examinations repeated at short intervals.

As an occasional consequence of long-continued nervous congestion of the uterus, Dr. Hodge enumerates simple hypertrophy, without inflammation or any change of tissue other than that of increased bulk.

Another very common result of the congestion incident to irritable uterus, according to our author, is *menorrhagia* or *hemorrhagia uteri*. This, by recurring at short intervals, by its profuseness, or by its long continuance, often renders the patient feeble, anæmic, dyspeptic, and even dropsical; while the cerebro-spinal system becomes so sensitive, so irritable, that both mind and body are reduced to a state of exhaustion. The entire organism acquires a morbid sensibility, and a horrible train of physical, mental, and moral disturbances set in, which render existence scarcely tolerable.

*Leucorrhœa* is also, according to Dr. Hodge, a frequent result of simple congestion in cases of irritable uterus, as a mere profluvia from the lining membrane of the uterus, altogether independent of inflammation. That such a form of leucorrhœa does occur we are not inclined to deny, and yet we think the rationale of it given by Dr. Hodge is, to say the least, of very doubtful accuracy. He supposes that an irritation is present similar to what exists at the menstrual nisus, and that this is followed by congestion and increased secretion. The leucorrhœa in these cases he views, therefore, as an imperfect menstruation—the fluid discharged not being fully elaborated—it is in fact, strictly speaking, the "white menses" of old authors.

Besides the simple, neuralgic form of dysmenorrhœa already referred to as a symptom of irritable uterus, Dr. Hodge admits three other forms of painful menstruation: 1st. The *congestive*. The normal vascular excitement of the uterus is aggravated; the fullness of the blood-vessels, at the catamenial period, becomes preternatural—inordinate. The nervous symptoms are augmented and often become very intense, and apparently threatening; severe sympathetic disturbances of the brain, spinal marrow, stomach, and bowels often follow. These, however, are usually of short duration, lasting sometimes for only two or three hours. 2d. *Mechanical dysmenorrhœa*. This is frequently due to obstruction of the canal of the cervix uteri, from the presence of coagula, inspissated mucus, lymph, or a membrane in the cavity of the neck or body of the womb; from turgescence or thickening of the lining membrane of the organ; the formation of strictures; or, finally, from the presence of a flexion of the cervix uteri, which our author believes to be the most frequent cause of obstruction. In some very rare cases, there is congenital contraction or deformity of the cervix. All these obstructions give rise to spasmodic efforts of the uterus for their removal. 3d. *Membranous dysmenorrhœa.* In this form of painful menstruation, there is either the formation of a deciduous membrane within the uterus, caused by the exfoliation of the proper mucous membrane of the womb, or by a desquamation of epithelial scales produced in such abundance as to present a membranous appearance—the membraniform formation in either case being discharged during menstruation, generally in large shreds, sometimes, however, in the form of a cast of the uterine cavity. It is evident that in no case are these membraniform substances the product of an inflammatory process. They differ from lymph in all their characteristics. Dr. Hodge ascribes their production to a high degree of nervous irritation, giving rise to congestion, followed by a kind of hypertrophy of the lining membrane of the uterus, with exfoliation.

The complications of irritable uterus—endometritis, metritis, tumors, etc., are the subjects embraced in the fourth chapter. A contrast is presented between the inflammatory conditions of the mucous tissue of the uterus and vagina and those of the substance and peritoneal surface of the womb, showing that the symptoms of the former are less intense and the disease far more tractable. Even in those chronic inflammations of the cervix involving the whole substance of the neck, with the granular appearance of its extremity, and the everted, tense, tumid lips of the os, few symptoms, Dr. Hodge remarks, exist to advertise the patient of her condition, and very often no general disturbances are excited. The patient often feels well. The reputed intractability of these inflammations, he believes, often arises from their complication with displacements, which keep up the irritation.

The *general* symptoms of irritable uterus are considered by Dr. Hodge: 1st. As cerebro-spinal irritations; giving rise to hyperæsthesia of the surface; irritations along particular nerves; convulsions, delirium, and various intellectual and moral disturbances. 2d. Reflex influences of cerebral and spinal irritation, upon the respiratory organs, the heart, the mammæ, the viscera of the abdomen, etc., giving rise, in different cases, to various disturbances of each of these portions of the body often simulating the most alarming of the organic diseases to which they are subject.

The *progress* and *results* of irritable uterus are next considered, under the heads of irritable rectum, irritable vulva and vagina, irritable bladder and urethra, morbid states of the ovaries, irritable lymphatic glands, and irritation of the pelvic nerves.

Of the author's teachings in reference to the several subjects embraced in the chapters we have thus briefly run over, we should be pleased to present a very full analysis, could we with propriety occupy the necessary space. To the work itself we must refer the reader; who will find, in the graphic description given of the various affections esteemed by Dr. Hodge as of a purely nervous origin—that is, dependent upon an irritation confined strictly to the proper nervous tissue—much to instruct—much that is calculated to lead to a correct diagnosis, treatment, and prognosis. He may not be inclined to view with the author the nervous diseases incident to the female as pure examples of hysteria, using the term in the same sense as that given to it by the older writers—that is, as dependent upon a direct action from the irritable condition of the uterine organ upon the cerebro-spinal system or particular portions of it, or a reflected action through the latter upon the different tissues and organs of the body; still, he cannot fail to derive from the descriptions and rationale of morbid phenomena contained in the chapters before us important suggestions as well pathological as therapeutical in respect to some of the most common and painful affections of the female.

As *predisposing causes* of irritable, that is to say, the purely neurotic diseases of women, Dr. Hodge enumerates the nervous temperament, rheumatism and gout, the parturient state, displacements of the uterus, a warm climate, and the sedentary, indolent habits engendered by it. As *exciting causes*, he sets down inflammatory congestion, parturition, overlactation, nimia venus, mental and moral excitements, cold, great muscular efforts, pressure upon the uterus, external or internal; uterine diseases, such as hypertrophy, induration, tumors within or on the exterior of the organ, or imbedded in its substance, physometra, hydrometra, abscess, obstructions (partial or complete) of the cervix uteri, etc., and irritations of other organs.

Dr. Hodge believes that there are many reasons which explain the greater prevalence of nervous affections of the uterus and their more distressing character now than formerly. One of these is the greater development of the nervous temperament in the female, from the increased indulgences and luxuries of modern female life, in which every stimulus is supplied adapted to procure a precocious development of the mind, the heart, and the passions, with an entire neglect of physical education, all which is adapted to unduly and too rapidly excite the animal at the expense of the organic life. Another reason is the improper style of clothing of modern times, with its tight lacing, its weight, its braces, etc. Dr. Hodge believes that upon inquiry, it will be found that the increased frequency of uterine diseases is, however, more apparent than real; these complaints passing formerly under a variety of names, leading the mind away from their actual nature and seat. Even at present, he thinks that the uterus, as a source of spinal and cerebral irritation, of neuralgia, spasms, and convulsions, is too often ignored.

It will be impossible for us to present any more than a very brief notice of the interesting and able account given by Dr. Hodge, of the treatment of irritable uterus. The first indication he lays down is, of course, the removal of the cause, whatever that may be, and whenever it is under the control of the physician. In fact, the treatment demanded for the removal of the cause is, in most cases, pretty much all that is required for the recovery of the patient.

The second indication to be fulfilled is to diminish or destroy the morbid irritability, whether general or local. This is to be attempted by two classes of remedies. The first includes all those agents which have a direct sedative or anodyne influence on the nervous system, particularly the antispasmodic and narcotic medicines, so useful as temporary means for assuaging pain and moderating uncomfortable nervous sensations. These are valuable as palliative measures, but seldom useful as permanent remedies. The second class of remedies, the indirect, are those which give strength, and impart vital vigor, and in this manner render the whole constitution less excitable, less irritable. These are a proper course of hygienic measures—a proper regulation of diet, of clothing, and of exercise and rest; exposure to a fresh and pure air; the external use of water, either generally or locally, and of such a temperature as the condition of the patient's constitution shall warrant; injections of cold water into the vagina or rectum.

In facilitating the reinstatement of the strength and vigor of the constitution, Dr. Hodge believes that much may be done by a judicious employment also of general and local medicinal agents. In acute cases circumstances may demand, for the relief of excessive congestion, at particular periods, and in women of full habits, the loss of blood, especially by cups or leeches to the sacral, hypogastric or iliac regions, the inside of the thighs, or to the pudendum. Revulsives, as hot baths, hot hip baths, warm poultices and fomentations, large, warm mucilaginous or watery vaginal injections, sitting over the vapor of hot water or herbs, he describes as particularly soothing and palliative. To relieve acute attacks of pain and spasm, narcotics are prescribed, either internally or locally, in poultices, or by friction, or by injection into the vagina or rectum. In chronic cases the loss of blood is of very doubtful propriety. The local application of warm water as above, though always palliative and grateful, should not, however, be persisted in for too long a period. The local application of cold water is perhaps the remedy from which the best effects are to be anticipated in the reduction and removal of excessive debility. It is recommended to be employed in the form of douches to the back, loins, and sacrum, or of sponging to the abdomen, thighs, and pudendum, followed by frictions of the surface. Tepid, cool, or cold vaginal injections, according to the sensations and experience of the patient, Dr. Hodge considers to be advantageous in the intervals of the catamenia. Cold rectal enemata he also esteems beneficial, when, especially, there is constipation.

Dr. Hodge thinks highly of the direct application of narcotics to the uterus, in the form of injections or suppositories. As occasional remedies, anodyne rectal enemata are pronounced by him to be very efficient.

Of the extremely popular remedy, the application of nitrate of silver to the cervix and os uteri, whatever advantages it may possess in cases of decided inflammation of these parts, Dr. Hodge believes that in pure cases of irritable uterus, while it may at first diminish morbid sensitiveness, it seldom affords permanent relief. In respect to the proposition to introduce bougies into the neck and body of the uterus with the view of blunting in this manner the morbid sensitiveness of the organ, he speaks in no very encouraging terms.

In the treatment of the *painful menstruation* consequent upon irritable uterus, while during the intervals we are to direct our attention to the correction of the local cause, whatever this may be, but especially to the replacement of the uterus in its proper position, and to the removal of obstructions of the cervix, by pessaries, sponge tents, bougies, etc., and by the enforcement of the whole treatment indicated for irritable uterus, during the paroxysm our chief object should be the palliation of the patient's suffering. Her bowels are to be opened by castor-oil or an enema, a hot pediluvium, hip or general bath is to be administered, and patient put to bed, with poultices or fomentations to her hypogastrium, hot applications to her feet, warm mucilaginous vaginal injections being given, followed by hot drinks and diaphoretics. When the pain is severe, narcotics may be administered. In the use of these latter, the utmost caution is to be observed. They are always to be viewed as temporary remedies only; their habitual use is always to be deprecated.

In cases of *menorrhagia*, while the general health of the patient is reinstated and sustained by a suitable hygienic and remedial treatment, Dr. Hodge insists that the main object should be the removal of the local irritation and congestion. Inflammation, if present, will demand mild local applications; displacements of the uterus require the use of an appropriate pessary. When the discharge is severe or persistent, a tonic and even stimulant treatment may in some cases be demanded, with astringents and ergot internally, and cold applications to the hypogastrium, rectum, and vagina, and astringent washes to the vagina and cervix uteri; and in obstinate cases, injections of tepid water or weak solutions of alum or sulphate of zinc, or some other astringent, may be thrown into the cavity of the uterus.

In cases of *leucorrhœa* the same practice, according to our author, is proper as in cases of chronic menorrhagia. Remove, he remarks, the irritable condition of the uterus, and you will cure the leucorrhœa. When displacements are present, as is generally the case, pessaries are the most efficient remedies: by relieving the irritation, they of course remove also the congestion and the discharge. As palliative measures, all the usual general and local treatment for anæmic and exhausted cases of irritable uterus will be found important in the management of leucorrhœa.

Some very important hints in respect to the character, effects, and treatment of enlarged and indurated uterus, uterine and ovarian tumors, will be found in the same chapter in which the therapeutics of the maladies above noticed is discussed.

In the treatment of the complications of irritable uterus, its secondary and sympathetic affections, Dr. Hodge lays down two leading indications: First, to detect and remove the cause, which, with him, constitutes very properly the radical treatment of all spinal and cerebral irritations; and, second, to diminish the morbid irritability of the cerebro-spinal system, by imparting tone to the whole body. These two indications cover the entire field of therapeutics applicable to the mere nervous affections—the *simulative* complaints—of the brain, larynx, lungs, heart, stomach, bowels, ovaries, uterus, etc. The prognosis in all cases of purely irritable diseases, he insists, is almost always favorable; nevertheless, it is admitted that in some few protracted cases, when improperly managed, or absolutely neglected, secondary complaints of a dangerous character may ensue.

Dr. Hodge assumes as an established fact, that the true proximate cause of the nervous diseases of women is in the greater number of instances an irritable uterus—it being usually, in such cases, "*ipse morbus*," the disease itself. Now, this irritable condition of the uterus he supposes most frequently to result from displacement of the organ; or when the latter does not actually produce it, it causes by its presence a persistence of the irritable condition, even after its original cause has been completely removed. With such views, it is evident that malpositions of the uterus and their treatment are with him subjects invested with no trifling degree of importance, their investigation being essential, in order to complete the analysis of the pathology and therapeutics of the state of morbid irritability of the organ, and of the neurotic diseases generally of the female constitution. Part *second* of the treatise before us is, therefore, devoted expressly to the consideration of uterine displacements, their varieties, causes, symptoms, and treatment.

Dr. Hodge describes first the natural position of the uterus, and the nature of its supports. The position of the uterus in the pelvis, and the admirable provisions for its sustentation, are such that, in the healthy robust female, though engaged in active labor, and on her feet for many hours every day, its displacement is of very rare occurrence, or it may even occur to a certain extent without any bad effects resulting.

The varieties of uterine displacements are next very accurately described, and their predisposing and exciting causes investigated. Among the predisposing causes, Dr. Hodge enumerates relaxation of the uterine ligaments and fascia; increased size and weight of the uterus; distention of the abdomen; pressure upon the abdomen; leucorrhœa, etc.; and among the exciting causes, sudden or prolonged muscular effort; the development of tumors on the anterior or posterior portions of the uterus, and various other developments which cause by their presence pressure upon the posterior or anterior surface of the womb.

We cannot attempt to furnish an analysis of the symptoms indicative of uterine displacements. Dr. Hodge admits that in females whose nervous system is strong, but at the same time not readily excited, the uterus may become displaced with apparent impunity. They may feel well, and all their functions may be easily and comfortably executed. In general, however, displacements of the uterus give rise to more or less inconvenience, which is augmented in intensity when from any cause or concurrence of causes the uterus has become morbidly sensible. The general deduction, which Dr. Hodge believes to have been confirmed by his own long-continued experience, is that malpositions of the uterus are intimately connected with all the phenomena of irritable uterus, either as original or as aggravating causes. That until such malpositions are rectified, neither the morbid condition of the organ itself nor of the cerebro-spinal system can be removed. So long as the displacement is permitted to remain, dysmenorrhœa, menorrhagia, and leucorrhœa, the pelvic sufferings, the inability to walk or stand, the spinal and cerebral irritations, with all the occasional but terrible disturbances of the larynx, lungs, heart, stomach, liver, kidneys, and bowels—especially the rectum—can hardly be completely relieved, and sometimes not even palliated; while, on the other hand, if the displacement be removed, recovery will generally quickly ensue.

Dr. Hodge admits that many cases of cerebro-spinal irritation, or *hysteria*, as the disease is usually named, may occur without uterine displacement, or even in which there is no uterine irritation of any kind whatever. In addition to these cases, he has met with others in which the displacement had been completely relieved, while the local and general symptoms of irritation, although palliated, were still present. This persistence of the phenomena of irritation is very often owing to our ignorance of their local origin; they may result from other causes than a displaced uterus, and from an irritation seated in some other of the tissues in the sacral region.

In the treatment of the various uterine displacements there are, according to our author, four indications to be fulfilled. 1. To remove or palliate any existing causes. 2. To replace the organ. 3. To maintain it steadily in its natural position. 4. To strengthen its natural supports.

For the fulfillment of the third indication, no reliance is placed upon any other measures than the introduction of pessaries of suitable material, size, and form. These are considered essential for their perfect relief. By them it is maintained that the uterus may very generally be replaced and held in situ.

"That the local symptoms of weight, pain, etc., the leucorrhœa, the menorrhagia, the dysmenorrhœa, and all the innumerable direct and indirect symptoms of spinal and cerebral irritation, including neuralgia, nervous headache, nervous affections of the larynx, lungs, heart, stomach, bowels, etc., as also spasms, cramps, and convulsions, may often be dissipated; that the intellectual and spiritual being may be elevated from the lowest state of depression, bordering on melancholy, or be delivered from the highest degree of maniacal excitement, and that the whole economy may thus be revolutionized. Patients often are amazed at their own altered sensations; they can hardly realize their identity—feeling as if they were either renovated, or that they had been transported to a 'new world.'"

After this eulogium upon pessaries—and no higher we believe has any one of their advocates ever ventured to pronounce—a deep interest will necessarily be felt to know the particular material and forms of pessaries which Dr. Hodge has found from experience to be best adapted to the several varieties of uterine displacement met with in practice. For the necessary information on this point we must refer our readers to the work before us. The lever pessary is the one preferred by him; though he admits that it acts more slowly in the restoration of the replaced organ than the intra-uterine pessary, yet, eventually, it is equally certain. At the same time,—

"It does not necessarily produce any irritation, organic or nervous, or any leucorrhœa, menorrhœa, inflammation, etc. It can be worn at all times, night and day; it interferes with no motion, and no function; the patient has no attention to pay to it, except a daily vaginal wash; she may, and often does forget its presence; can enjoy her connubial pleasures, can move about in society without anxiety, is free from local and general nervous irritations, from corporeal, intellectual, and spiritual disturbance; and the physician may hope that the uterus being perfectly sustained, the ligaments, now free from every counteracting influence, will continue to contract to their normal length, and acquire their original tonicity, so that a permanent cure may be effected, or that pregnancy ensuing, the continued use of the pessary will preserve his patient from those irritations so frequently excited by displacements, and so apt to result in abortions."

When, in support of the foregoing statements, we have adduced the experience of a practitioner of the age and standing and cool inquiring mind of Dr. Hodge, we are scarcely warranted in doubting their accuracy. It is true, we have the admission, that it is not in every case of uterine displacement we are to expect from the use of pessaries complete success; partial relief is sometimes all that can be obtained. The difficulties Dr. Hodge believes to arise from the improper character of the instruments employed, the mechanical obstructions to a replacement of the organ, the great sensibility of the vital tissues involved, the timidity and nervousness of the patient, and too frequently from the want of proper indications in carrying out the treatment.

For ourselves we are not at all surprised at the acknowledgment of the small amount of relief often obtained from the use of pessaries in cases of irritable uterus; our wonder is that with the morbid conditions so well described by our author, they should ever succeed; that they do not, in fact, in every instance in which they are resorted to aggravate the sufferings of the patient. We confess we cannot understand how it is possible, when the uterus, the vagina, the bladder, and the rectum are all in a state of the most intense morbid sensibility, the introduction of a metallic lever, adapted to force as it were, and sustain the dislocated and contorted and exquisitely irritable womb in its normal position, can afford the great and prompt relief ascribed to it. That such relief does result from its use is proved, it is said, by the large and carefully analyzed experience of Dr. Hodge. While we admit the very great weight of the evidence thus adduced, may we not be permitted, without offending, to suggest that experience may sometimes be fallacious? We firmly believe that far too much importance has been ascribed to slight displacements of the uterus. It is our opinion that the irritable state of this organ is not always nor very generally due to local causes acting directly upon the organ itself, but is the result of the general morbid irritability of the system at large. Let this latter be removed by an appropriate hygienic and medical course of treatment, and the irritability of the uterus will be cured in despite rather than by the aid of pessaries.

In relation to tumors of the uterus which occasionally complicate displacements of the organ, Dr. Hodge is particularly decided in his opposition to the vaginal operation for their enucleation, as altogether useless in cases even of apparent or reputed success. He believes, besides, that they must be often injurious not merely from the pain and irritation they produce, the inflammation and abscesses which may be their consequences, but from the danger, also, of their facilitating the development of any tumor which may remain, and any malignant predisposition to which it may be liable. Nearly the same objections (with the additional one of the danger of peritoneal inflammation) hold good, in his opinion, against the attempt to remove these tumors by an abdominal section. Equally opposed is our author to the operation of ovariotomy. His chapter on enlargements and displacements of the ovaries demands a careful perusal on the part of every practitioner; it is rich in facts and replete with instruction.

Part *third* of the treatise is devoted to a brief consideration of the diseases of sedation, or those dependent upon a condition of a part or of the whole system, directly opposed to excitement or irritation; a condition not of diminished vital power but of diminished action. It may result from irritations in a part or organ of the system, of which sedation in another part or organ is a common consequence; from previous inordinate excitement, great muscular effort, the indulgence of animal passions, or from the relaxing effects of heat with or without moisture. Or it may result from mechanical causes, as severe contusions or concussions; from the abstraction of the natural stimuli—caloric, as it influences a portion or the whole of the body; oxygen in respect to the lungs; food in respect to the stomach; bile in respect to the intestines; mental and moral sentiments in respect to the brain, etc. Or, finally, it may be induced by certain medicinal agents, sedatives, narcotics, antispasmodics. The leading effect of sedation is the diminution or suspension of the proper functions of the tissue or organ involved. Applying the pathological views of sedation developed in the first chapter of the closing part of the treatise, especially to the womb and its appendages, Dr. Hodge is led to the consideration of its most prominent phenomenon, amenorrhœa—the non-appearance or arrest of the menses—a phenomenon that is in every case in which it occurs simply a sign—a symptom of abnormal states of the uterus, of the ovaries, of the secretory organs, or of the general nervous and vascular systems, by the removal of which alone, when remediable, can the menstrual flux be established or reinduced.

The whole subject of sedation, whether in its general aspect, or in reference especially to the uterus and its associated organs, is one replete with the deepest interest and importance. In regard to it opinions the most erroneous have been entertained, that have led to mistakes in practice of a very grave character; we would recommend an attentive study of the views presented in respect to it in the treatise under review. They bear the impress of truth, and will, we believe, be found trustworthy guides to the proper therapeutical indications in all the diseases of sedation, but more especially to that dependent on sedation of the uterine organs.

What gives to the treatise of Dr. Hodge its chief value, and will cause it to be received as an original contribution to pathology and therapeutics, is not so much its teachings in respect to uterine displacements and their treatment, as the doctrines it announces in respect to the nature and character of the purely nervous affections, irritable diseases, and nervous irritations; showing that they are not the mere conceptions of a disturbed enervation, the appendage of some chronic phlegmasia of this or that tissue, but original maladies, having an actual existence and local habitation; being, in fact, physical and not solely moral disturbances, and demanding therefore for their removal physical as well as moral remedies.

